# Correlation of Prehypertension with Left Ventricular Mass Assessed by Cardiac Magnetic Resonance Imaging

**DOI:** 10.1155/2015/742658

**Published:** 2015-10-12

**Authors:** Tarek M. Mousa, Oluwaseun A. Akinseye, Ketevan Berekashvili, Olakunle O. Akinboboye

**Affiliations:** ^1^Queens Heart Institute, 234 36 Merrick Boulevard, Laurelton, NY 11422, USA; ^2^Department of Medicine, Icahn School of Medicine at Mount Sinai, Queens Hospital Center, 82 68 164th Street, Jamaica, NY 11432, USA; ^3^Department of Neurology, SUNY Downstate Medical Center, 450 Clarkson Avenue, Brooklyn, NY 11203, USA

## Abstract

*Introduction*. The purpose of this observational cross-sectional study was to assess left ventricular mass (LVM) in prehypertensive individuals in comparison to normotensives and to determine if central blood pressure (BP) correlates better with LVM index (LVMI) than brachial BP. *Methods and Result*. Brachial and central BP measurements were completed at first visit and at 4 weeks in 65 healthy volunteers who were at least 40 years old and not on medication. Subjects were divided into two groups of normotensives and prehypertensives based on JNC-7 criteria and LVM was obtained using cardiac magnetic resonance imaging. Prehypertensives had significantly higher LVMI compared to normotensives (*P* < 0.01). Brachial and central BP also both positively correlate with LVMI (*r* = 0.460, *P* < 0.01; *r* = 0.318, *P* = 0.012, resp.) in both groups and neither method was superior to the other. After multivariate regression analysis and adjusting for cardiovascular risk factors, prehypertension remained an independent determinant of LVM. *Conclusion*. Prehypertension is associated with cardiovascular target organ damage, and central BP was not superior to brachial BP or vice versa for association with LVMI.

## 1. Introduction

Prehypertension (PHT) was first introduced by the Seventh Joint National Committee on Prevention, Detection, Evaluation, and Treatment of High Blood Pressure (JNC-7) in 2003, replacing former categories of “high-normal” and “above-optimal” blood pressure (BP); and it was defined as systolic BP of 120–139 mmHg or diastolic BP of 80–90 mmHg based on 2 or more properly measured seated BP readings on each of 2 or more office visits [1]. The 2005-2006 National Health and Nutrition Examination Survey estimated 28% of United States adults had PHT [2]. Patients with PHT are at increased risk of developing hypertension and other cardiovascular diseases (CVDs) compared to normotensives [3].

PHT has been associated with significant abnormality of left ventricular (LV) geometry [4]. Although a study done by Zhu et al [5] did not find any statistical difference in the parameters of LV structure between normotensive and prehypertensive subjects, some studies have shown a linear correlation between PHT and increased left ventricular mass (LVM), with target organ damage found in both prehypertensive youths [6, 7] and older population [3, 4] when compared to normotensives. In fact, there has been increased risk of mortality associated with the prehypertensive category of BP [8]. The Prospective Studies Collaboration examined relationship between categories of BP and subsequent mortality by following almost 1 million people with no previous vascular disease prospectively for a total of 12.7 million person-years in 61 observational studies. They concluded that there is a continuous increase in mortality from both stroke and ischemic heart disease from BP of 115/75 mmHg, with a twofold increase in cardiovascular death in those with 20 mmHg higher systolic pressure or a 10 mmHg higher diastolic pressure, a level well within the range of PHT [8]. However, there is currently no indication for pharmacological treatment of this BP category.

Studies have shown that brachial and central BPs may differ, especially the systolic component [9, 10], but debates are still ongoing on which of the two correlates more strongly with left ventricular mass index (LVMI) and other CVD [11–13].

The aim of this study was (1) to assess the strength of association of brachial and central pressure with LVMI and (2) to compare these variables in prehypertensive and normotensive study participants. Cardiac magnetic resonance imaging (MRI) was used to diagnose LVM as it is more precise and reliable compared to other diagnostic modalities [14].

## 2. Methods

### 2.1. Study Design

We conducted an observational cross-sectional study of healthy volunteers from September 2008 to September 2009 at Cardiology Division of New York Hospital Medical Center of Queens/Weill Medical College of Cornell University. We recruited our participants using flyers distributed throughout our institution. We enrolled healthy volunteers who were at least 40 years old and not on medication. Exclusion criteria for the study were previous diagnosis of hypertension (HTN), diabetes mellitus (DM), renal disease, CVD, valvular heart disease, and atrial fibrillation.

Detailed history, physical examination, and brachial and central BP measurement were completed at the first visit. Height (m) and weight (kg) were measured and body mass index (BMI) was calculated as weight (kg)/(height (m) × height (m)) and unit was recorded as kg/m^2^. The second visit, which was 4 weeks after the first, included the second measurements of brachial and central BP and performing of a cardiac MRI. To ensure a more representative BP value for each patient, the average of the BP measurement at the first and second visit was used for analysis.

Study population was divided into two groups of normotensives and prehypertensives based on their BP measurement, and LVMI was compared in both groups. PHT as defined by JNC-7 criteria is systolic blood pressure (SBP) between 120 and 139 mmHg and diastolic blood pressure (DBP) between 80 and 89 mmHg. Brachial and central BP measurements with their correlation to LVMI were also studied.

The study was approved by Institutional Review Board of New York Hospital Medical Center of Queens.

### 2.2. Measurements of the Variables

#### 2.2.1. Brachial Blood Pressure

Brachial BP was measured with a BPM-300 noninvasive BP monitor (VSM MedTech Ltd., Vancouver, Canada) after the subject had been in a recumbent position for a minimum of 10 minutes. The device took 6 consecutive BP readings, excluded the first measurement, and derived an average. It uses an oscillometric technique to calculate systolic and diastolic BP. The device meets the Association for the Advancement of Medical Instrumentation Standards.

#### 2.2.2. Central Blood Pressure

SphygmoCor monitor was used to measure central BP. The tonometer, gently pressed against the radial artery pulse, acquires the radial pulse wave and the SphygmoCor's proprietary algorithm derives the pulse wave as it exists in the ascending aorta producing the central BP measurements noninvasively.

#### 2.2.3. Left Ventricular Mass

We used Siemens 1.5T cardiac MRI scanner to obtain LVM. We acquired ECG-gating and breath-holding during contrast-enhanced segmented* k*-space inversion-recovery with steady-state free precession imaging. LVMI was calculated using LV measurements in diastole, divided by height, squared, and expressed as g/m^2^.

### 2.3. Statistical Analysis

Continuous variables were summarized by the mean ± SD. Categorical variables were summarized by frequencies with 95% confidence interval (CI). Pearson *r* was used for correlations for each variable of interest (i.e., brachial SBP, central SBP, brachial DBP, central DBP, and LVMI). Means of continuous variables were compared with independent *t*-test statistics.

Multivariate linear regression models were constructed using PHT as the primary risk factor and effect size was adjusted for typical potential confounders (e.g., age, race, gender, BMI, and cardiovascular risk factors); *P* values ≤ 0.05 were considered statistically significant. Analyses were performed using SPSS statistical software (PASW statistics 18).

## 3. Results 

The study population (*n* = 65) consists of healthy volunteers (Table 1). The average age at the time of enrollment was 54 ± 8 (range from 43 years to 77 years); 65% were female; mean BMI (Kg/m^2^) was 27 ± 4 (range of 19–42). 29 (45%) of volunteers were prehypertensive and 36 (55%) were normotensive. 58.5% of volunteers were Caucasian, 27.7% were African Americans, 6.2% were Hispanic, and the rest were Asians. There was no statistically significant difference between prehypertensive and normotensive group in all of the following parameters: age, gender, race, BMI, augmentation pressure, augmentation pressure index, and central SBP.

**Figure 1:**
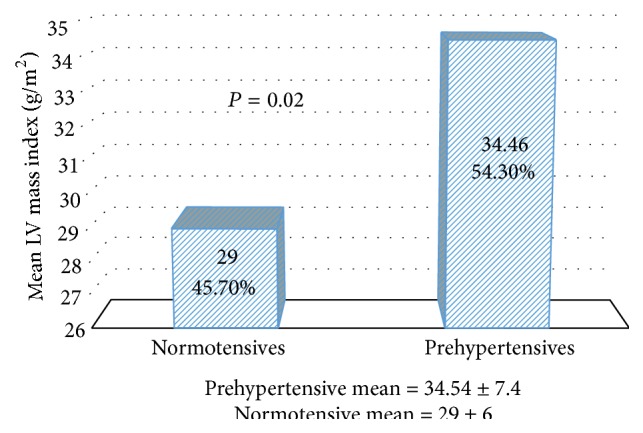
LV mass index in prehypertensive versus normotensive volunteers.

The main dependent variable we used in our statistical analysis was end diastolic LVMI (using height when calculating the index). Prehypertensives had higher LVMI that was statistically significant as compared to normotensives, *P* < 0.01 (Figure 1). Simple linear regression analysis showed that central SBP has positive statistically significant association with end diastolic LVMI with *P* = 0.014 (standardized beta coefficient = 0.314).

**Figure 2:**
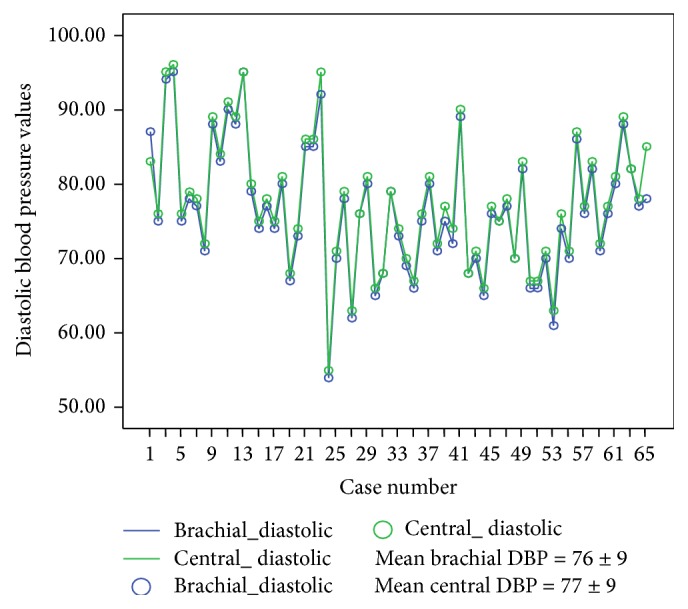
Brachial and central diastolic blood pressure measurements.

**Figure 3:**
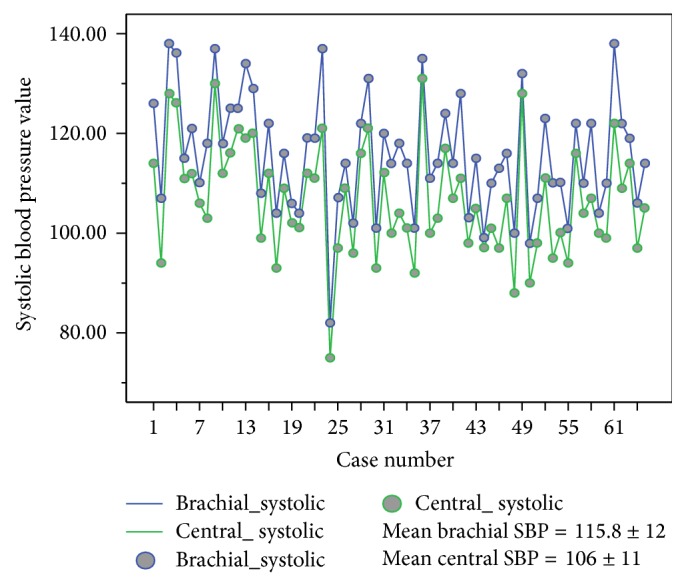
Brachial and central systolic blood pressure measurements.

There was no difference between brachial and central DBP (mean brachial DBP = 76 ± 9 mmHg versus central DBP = 77 ± 9 mmHg) (Figure 2); however, there was slightly higher brachial SBP (mean 115.8 ± 12 mmHg) compared with central SBP (mean 106 ± 11 mmHg) (Figure 3).

**Figure 4:**
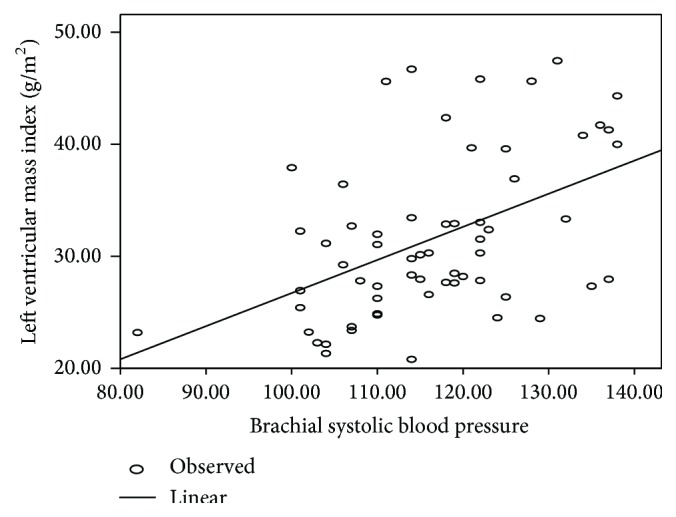
Correlation between LV mass index (kg/m^2^) and brachial systolic blood pressure.

**Figure 5:**
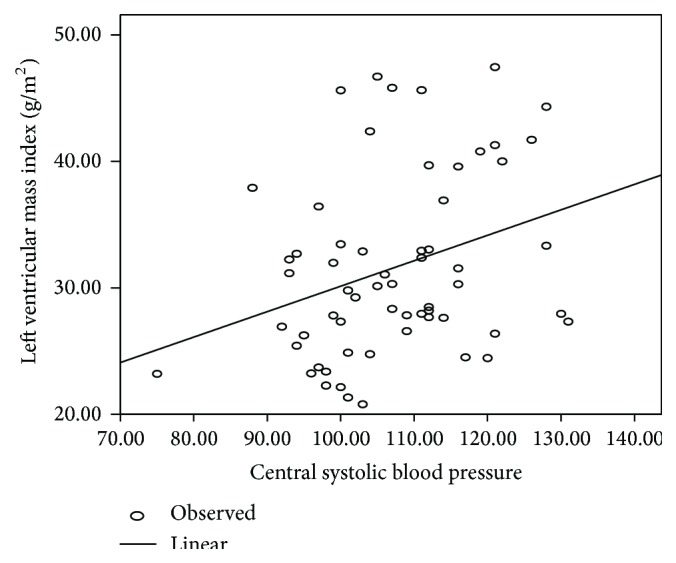
Correlation between LV mass index (kg/m^2^) and central systolic blood pressure.

Pearson's correlation showed statistically significant correlation between central SBP and LVMI (*r* = 0.318, *P* = 0.012) (Figure 4) and brachial SBP and LVMI (*r* = 0.460, *P* < 0.01) (Figure 5). The correlation coefficient between brachial DBP and LVMI (*r* = 0.521, *P* < 0.01) and central DBP and LVMI (*r* = 0.523, *P* < 0.01) was similar.

Multivariate linear regression analysis, when adjusted for age, gender, race, and BMI, showed positive statistically significant association between PHT and end diastolic LVMI.

## 4. Discussion

Prehypertensive individuals have been shown to be at an increased risk of developing hypertension [3]. Although hypertension is a well-documented independent predictor of elevated LVMI [6, 15, 16], few studies have shown the relationship between PHT and structural changes in the LV.

Our study demonstrated a strong relationship between PHT and LVMI when compared to normal BP, even after adjustment for age, gender, race, and BMI. Another principal new finding in present study was that, in both prehypertensives and normotensives, brachial and central BP correlated positively with LVMI, and central BP was not superior to brachial BP or vice versa for association with LVMI.

### 4.1. Prehypertension and Left Ventricular Mass Index

Manios et al. [17] analyzed the impact of PHT on LVM. They found a statistically significant association between prehypertensives and LVM (*P* = 0.03) compared to normotensive patients after adjustment for baseline characteristics. Our study supports this finding. We were able to establish the importance of PHT category to the increased risk of developing future CVD.

Left ventricular hypertrophy (LVH), measured by LVMI, has been identified as the most powerful risk factor for future cardiovascular events causing morbidity and mortality [15]. In fact, Richey et al. [18] studied the relationship between ambulatory BP and increased LVM in children at risk for hypertension and found that the odds ratio (OR) of having elevated LVMI increased by 54% for each incremental increase of standard deviation score (SDS) in 24-hour systolic SDS after controlling for race and BMI (OR = 1.54, unit = 1 SDS, CI = 1.1, 2.15, and *P* = 0.011) and increased by 88% for each increase of 0.1 in BP index (OR = 1.88, CI = 1.03, 3.45, and *P* = 0.04).

PHT is associated with an increased prevalence of LVH [4, 17]. In the Bogalusa Heart Study, Toprak et al. [19], in addition to finding a significantly higher LVMI in prehypertensives compared to normotensives, a finding supported by present study, also found PHT was significantly higher among men than women (35% versus 22%) and among blacks than whites (29% versus 27%). We did not find any significant difference between prehypertensive and normotensive groups by gender or race. The difference between both studies on the significance of race on BP category might be due to the smaller sample size (*n* = 65) of our study compared to the Bogalusa Heart Study (*n* = 1379). The gender differences might be explained by the age difference in both study populations. The average age of present study population was 54 ± 8 (43–77 years) as compared to the Bogalusa Heart Study with age range of 20–44 years. Arterial stiffening increases in both genders with age [20] which may explain why gender was not statistically significant in our prehypertensive group but was in the earlier study which was conducted in a much younger population. However, a recent study that analyzed sex differences in arterial stiffness and ventricular-arterial interactions, done in older population (men 67 ± 9 and women 65 ± 10), showed women had greater aortic stiffening as evidenced by higher aortic characteristic impedance (Zc) which should translate into a greater increase in BP from increased flow during LV ejection [21]. This motivates further research to determine the impact of gender on Zc and its role as a risk for developing PHT and future CVD.

Recent study on 1,940 young participants found higher LVM values in prehypertensives compared to normotensives even after adjustment for covariates [6, 17]. In contrast, Zhu et al. [5] did not find any statistical differences in LVMI between prehypertensives and normotensives. Methodological differences (such as age of study population, inclusion criteria, and ambulatory blood pressure measurement protocol) between these studies may have accounted for the different results.

Elevated LVM is a well-defined independent modifiable risk factor for adverse cardiovascular event [7, 15, 22] and for developing hypertension [7]. In fact, as reported by Urbina et al. [7], the progression of PHT to sustained hypertension was predicted by baseline systolic BP and baseline LVM, with the probability of developing hypertension increasing by 36% for each standard deviation of LVMI. This describes a vicious cycle in which PHT causes elevated LVM which in turn accelerates the progression of PHT to sustained hypertension increasing future risk of CVDs and mortality. Our findings confirmed that subjects with PHT have significantly higher LVMI than normotensives.

### 4.2. Central versus Brachial Blood Pressure and Left Ventricular Mass Index

Our study showed no difference between the diastolic components of both brachial and central BP but a slightly higher brachial SBP than central SBP (Figures 2 and 3). This supports the finding of a cardiovascular physiology study which showed that, for the same mean arterial pressure, SBP and pulse pressure (PP) are higher in peripheral (brachial) than in central arteries (thoracic aorta, carotid arteries) [23]. The difference, called SBP or PP amplification, is a result of the progressive reduction of the diameter and increase in stiffness from the proximal to the distal arterial vessels and mostly of the modification in the transit of wave reflections [23, 24]. However, even though brachial and centrally measured SBP differ, our observation supports previous evidence that both central and brachial SBP positively correlate with LVMI in normotensive and prehypertensive individuals [11, 12, 25]. The central BP was measured using SphygmoCor monitor which has been shown to have excellent interobserver reproducibility [9] which accords with that reported by other workers using different methodologies [26].

There is an on-going debate on the BP approach that correlates better with LVMI and CVD. Roman et al. [25] reported that LV relative wall thickness and mass index were more strongly related to central than brachial BP, so were the findings in other studies [11, 12]. Na et al. [12] concluded that central BP, measured as central PP, was a stronger predictor of LVMI than peripheral PP (*β* coefficient = 0.311, *P* = 0.001 versus *β* coefficient 0.281, *P* = 0.003 resp.). Pini et al. [27] also suggested the superior prognostic utility of central BP compared to brachial BP in an unselected geriatric population. In contrast, Dart et al. [13] found that brachial BP but not central BP had a better prognostic impact. A new finding in our study is the nonsuperiority of central BP to brachial BP in correlation with LVMI or vice versa. To the best of our knowledge, this is the first study reporting this finding. Further prospective studies are required to determine whether central BP may be a better predictor of LVMI and future CVD and mortality.

## 5. Conclusion

In conclusion, this data provides evidence of increased LVMI in prehypertensive patients. An increased LVM has been shown to be an independent modifiable risk factor for adverse cardiovascular events and progression of PHT to sustained hypertension. Current guidelines recommend lifestyle modification for the management of PHT, but this has had no demonstrable effect on public health to date [28]. The Trial of Preventing Hypertension (TROPHY) study demonstrated for the first time that pharmacological treatment of prehypertensives was safe and partially reduced the risk of developing incident hypertension; however, no difference in the occurrence of cardiovascular events was observed between the treatment groups [28]. We recommend further prospective studies to determine whether pharmacological treatment of prehypertensives provides a cost-effective strategy for reducing CVD risks.

In addition, both central and brachial BP positively correlate with LVMI in normotensive and prehypertensive patients and central BP was not superior to brachial BP or vice versa for association with LVMI.

## Limitations

Our study population consists of relatively small sample of 65 volunteers which may limit the generalizability of the results. There is need for further studies with larger sample populations to replicate these findings. Furthermore, the average age of our study population was 54 ± 8, with older age individuals underrepresented; however, PHT remained an independent predictor of increased LVMI even in multivariable models where age was entered as covariate.

BMI was indexed to body surface area (BSA) (kg/m^2^) in our study; however, there is on-going controversy on the best method to index LVM so as to account for body size. Indexing LVM to BSA is said to underestimate LVM in obese and overweight hypertensive patients when compared to height^2.7^ indexed LVM [29]. Indexing LVM to BSA in present study showed that PHT is associated with LVMI, and this association could have been stronger if LVM was indexed to height^2.7^ as we might have underestimated the prevalence of increased LVM in our study population by using LVM indexed to BSA.

## Figures and Tables

**Table 1:** Patient's characteristics, left ventricular mass index, and blood pressure categories.

	Prehypertensives	Normotensives	Statistical significance
	Yes (*n* = 29) 45%	No (*n* = 36) 55%	
Age (mean ± SD)	56 ± 9.6	52.8 ± 7.3	0.122
Race (number/%)			
White	17 (58.6)	21 (60)	Not significant
Black	8 (27.6)	10 (28.6)	
Asian	3 (10.4)	1 (2.9)	
Hispanic	1 (3.4)	3 (8.5)	
Gender (number/%)			
Female	18 (62.1)	24 (66.7)	0.796
Male	11 (37.9)	12 (33.3)	
BMI (kg/m^2^)	27.5 ± 4.5	26.7 ± 3.8	0.4
BSA (m^2^)	1.88 ± 0.26	1.85 ± 0.18	0.57
Augmentation pressure (mmHg)	8.4 ± 5.6	6.3 ± 4.4	0.096
Augmentation pressure index (%)	23.8 ± 13	20.11 ± 14	0.275
LV mass index (g/m^2^)	34.45 ± 7.4	29 ± 6	0.002

BMI: body mass index, BSA: body surface area, LV: left ventricle, and LVMI: left ventricular mass index.

Values are presented as mean ± SD.
